# Terminology integration and inconsistency identification of adverse event terminology for Japanese medical devices using SPARQL

**DOI:** 10.1186/s12911-022-01748-2

**Published:** 2022-01-19

**Authors:** Ayako Yagahara, Hideto Yokoi

**Affiliations:** 1 Faculty of Health Sciences, Hokkaido University of Science, 7-Jo 15-4-1 Maeda, Teine, Sapporo, Hokkaido 006-8585 Japan; 2 Faculty of Health Sciences, Hokkaido University, Sapporo, Hokkaido Japan; 3 Department of Medical Informatics, Kagawa University Hospital, Kita-gun, Kagawa Japan

**Keywords:** Terminology mapping, Inconsistency detection, Medical device, Adverse event, Resource Description Framework, SPARQL Protocol and RDF Query Language

## Abstract

**Background:**

For standardization of terms in the reports of medical device adverse events, 89 Japanese medical device adverse event terminologies were published in March 2015. The 89 terminologies were developed independently by 13 industry associations, suggesting that there may be inconsistencies among the terms proposed. The purpose of this study was to integrate the 89 sets of terminologies and evaluate inconsistencies among them using SPARQL.

**Methods:**

In order to evaluate the inconsistencies among the integrated terminology, the following six items were evaluated: (1) whether the two-layer structure between category term and preferred term is consistent, (2) whether synonyms of a preferred term are involved. Reversing the layer-category order of matching was also performed, (3) whether each preferred term is subordinate to only one category term, (4) whether the definitions of terms are uniquely determined, (5) whether CDRH-NCIt terms corresponding to preferred terms are uniquely determined, (6) whether a term in a medical device problem is used for patient problems.

**Results:**

About 60% of the total number of duplicated terms were found. This is because industry associations that created multiple terminologies adopted the same terms in terminologies of similar medical device groups. In the case that all terms with the same spelling have the same concept, efficient integration can be achieved automatically using RDF. Furthermore, we evaluated six matters of inconsistency in this study, terms that need to be reviewed accounted for about 10% or less than 10% in each item.

**Conclusions:**

The RDF and SPARQL were useful tools to explore inconsistencies of hierarchies, definition statements, and synonyms when integrating terminolgy by term notation, and these had the advantage of reducing the physical and time burden.

## Background

It is important to collect and analyze reports of adverse events of medical devices to be able to improve the safety of medical devices. To achieve this, and especially to identify causes of adverse events and patient problems, it is necessary to use statistical analysis to standardize the terms used in reports, to establish information that will be helpful to improve the safety of medical devices.

For the standardization of terms, The Ministry of Health, Labour and Welfare of Japan announced official terms for use with Japanese medical devices in the event of adverse events in March 2015 [1]. These include 89 terminology items for groups of Japanese medical device nomenclatures developed by 13 industry associations (Table 1) who are members of The Japan Federation of Medical Devices Associations (JFMDA), and these 89 terminology items are collectively named the JFMDA terminology. Internationally, The International Medical Device Regulators Forum (IMDRF) was conceived to accelerate international medical device regulatory harmonization and convergence in 2011 [2].

**Table 1:** List of product group names (Excerpt)

Terminology ID	Groups in Japanese medical device nomenclatures
A01-1	Medical X-ray apparatus and relevant apparatus
B01-1	Biological information monitor—central monitor
B02-1	Ultrasonographic apparatus
D01-1	Anesthesia apparatus
D02-1	Artificial respirator
D03-1	Electrosurgical devices
D04-1	Ultrasonic surgical device
D05-1	Bag valve mask

At the same time, the 13 Japan industry associations independently each developed the terminologies using a bottom-up approach by gathering the terms used regularly in medical facilities to facilitate communication between medical staff and medical device manufacturers. Therefore, there may be inconsistencies among these terminoloigy items. A previous study stated that inconsistencies in terminology have a negative impact on effective communication and making sense of research findings, integrating studies, and building an integrated theory [3]. Conclusively, any heterogeneity of the JFMDA terminology may lead to inaccurate report analysis and interpretations of results. This makes it necessary to perform an accurate and detailed auditing of the terminology. Since there are few experts for auditing terminology in the JFMDA, it is to provide a continuous cycle including the automatic creation of inconsistency lists and considerations for terminology improvement.

In addition, several methods have been proposed for auditing inconsistencies in terminology. Van Damme et al. [4] evaluated the correctness and completeness of the ontology by referring to templates generated by lexical and clustering techniques. Bodenreider [5] describes the causes and solutions of Circular Hierarchical Relationships in UMLS. Zheng et al. [6] identified the missing is-a relationship using a transformation-based method to replace noun chunks in a concept name with more general concept names, and Cimino [7] visually detected inconsistent relations. However, various methods for auditing have been proposed, it is desirable to be able to automatically detect inconsistencies based on a policy of terminology creation to continuously maintain the terminology in a situation where there are few terminology specialists. In order to manage JFMDA terminology efficiently in that kind of situations, it is useful to describe the structure of the terminology by descriptive logic and detect inconsistencies using inference. Hoehndorf et al. [8] generated a relation template and detected inconsistencies of biomedical ontologies by inference. Jiao et al. [9] used the Resource Description Framework (RDF), which is a language for description logic to identify errors such as syntax errors and logical inconsistencies. Description logic has the advantage of maintaining the consistency of the structure in the terminology, and RDF is the standard model for data exchange on the Web and has been developed and agreed by the World Wide Web Consortium [10].

Our strategy was to integrate all terminology items and identify inconsistencies in hierarchical structures with an automatic approach. Because there are about 3500 terms used with problems generated by medical devices, manual verification requires considerable effort. It is necessary to develop tools to map these as described by RDF and identify inconsistencies using query templates created based on the policy of JFMDA terminology. In a previous study, we integrated 89 terminologies and determined their respective inconsistencies and focused on the terms expressed [11]. In this study, we focus on evaluating inconsistencies in a hierarchy structure and relationships between terms for the elaboration of the terminology. The purpose of this study was to enable evaluation of JFMDA terminology inconsistencies automatically using RDF and its query, SPARQL Protocol and RDF Query Language (SPARQL), from the point of view of hierarchy structure and relationships among terms.

## Methods

### JFMDA terminology

**Fig. 1:**
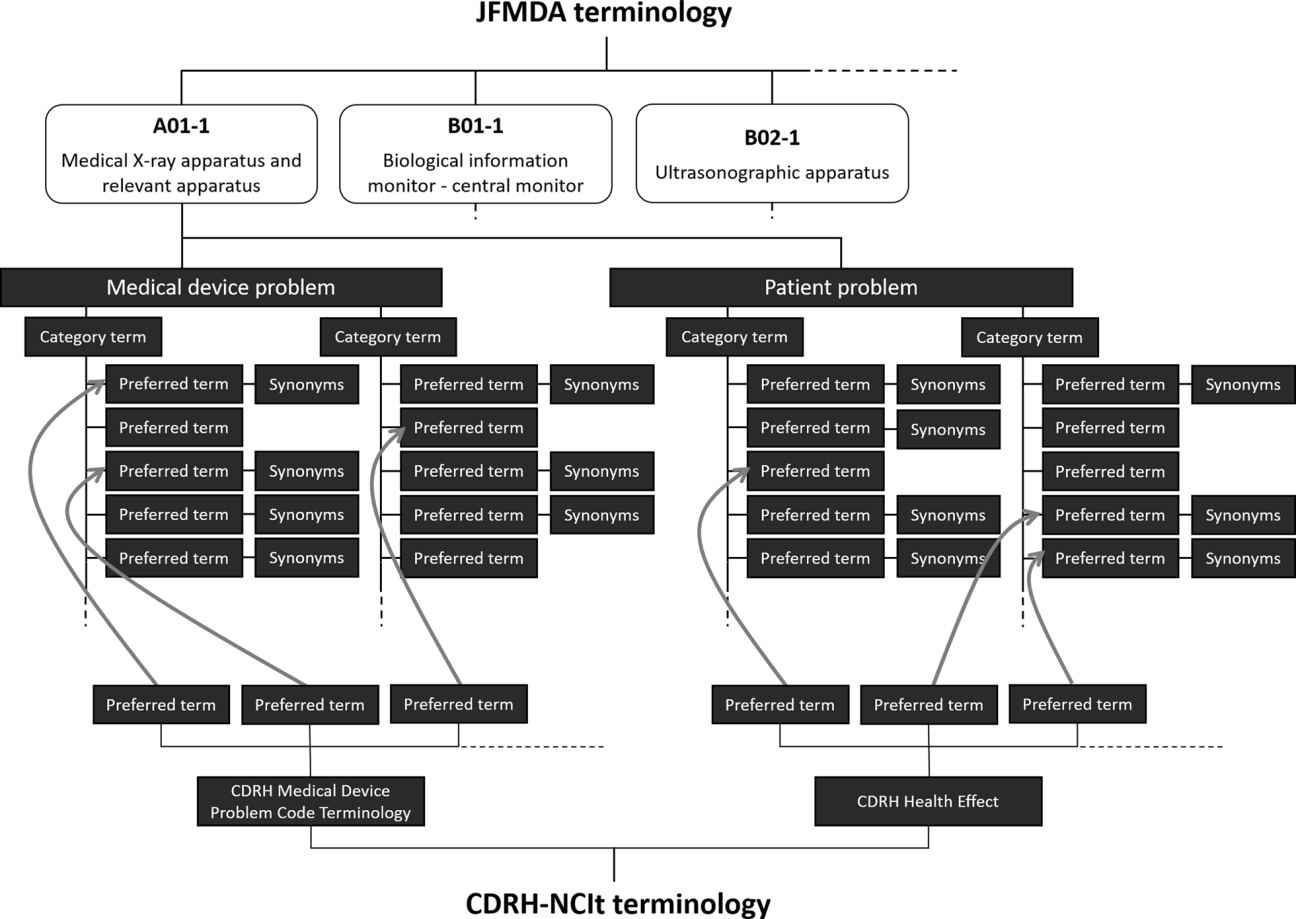
Hierarchical structure of JFMDA terminology 1st edition. Each terminology item has medical device problem and health problem

The JFMDA terminology 1st edition presented 89 terminology sets. Each terminology set includes the names of groups of terms used in Japanese medical device nomenclature, the industry associations that created the terminology, and the Japan medical device nomenclature which the terminology applies to. “Medical device problems” and “patient problems” in the terminology consists of “preferred terms” described as the detail of adverse event and patient problem and “category terms,” which categorized preferred terms into two hierarchies. Each “category term” has one or two or more preferred terms, and each “preferred term” has one or two or more synonyms. In addition, a preferred term is mapped a term for the centers for devices and radiological health and the national cancer institute's thesaurus (CDRH-NCIt) terminology [12] (Fig. 1). Table 1 shows examples of product group names. Incidentally the latest version is 4th edition and it has four categories of terms: “medical device problems”, “patient problems”, “investigation method, findings and conclusion” and “medical device component/accessary.” In addition, a preferred term is mapped as a term for the IMDRF code replacing the CDRH-NCIt [13].

### Terminology integration

**Fig. 2:**
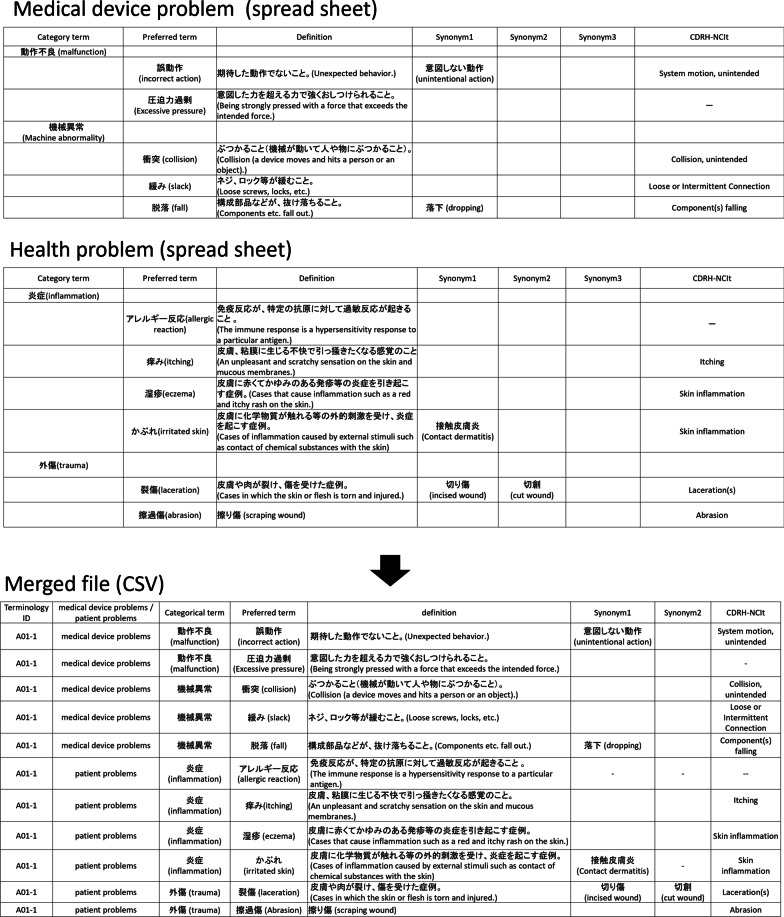
Conversion of spread sheet to CSV (English terms in parentheses)

**Fig. 3:**
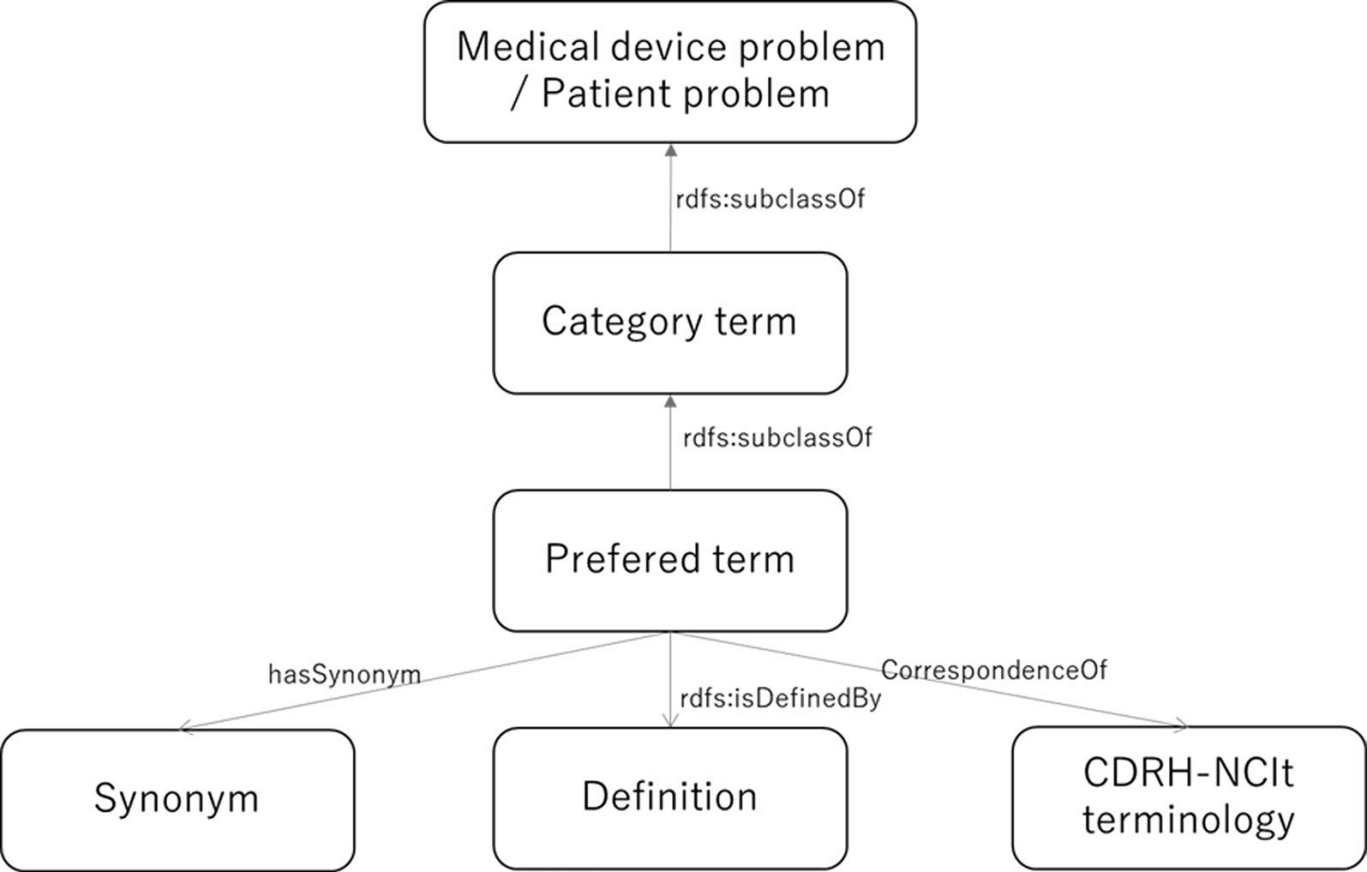
Architecture of the relationships in JFMDA terminology

**Fig. 4:**
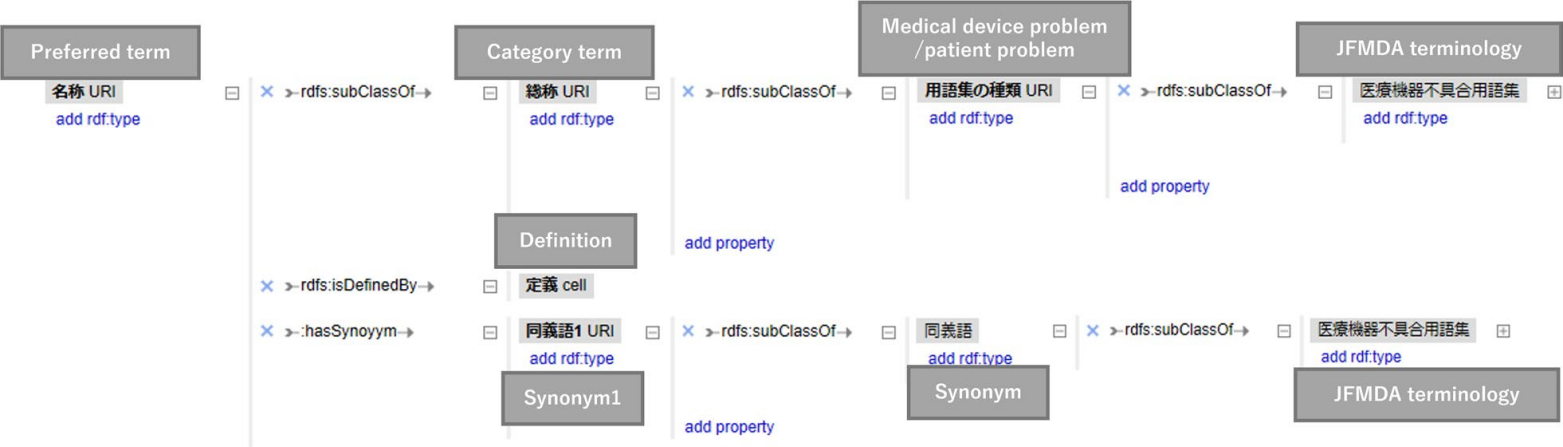
Conversion of CSV to RDF using Google Refine (Excerpt)

In this study, the first edition of the JFMDA terminology was used. The 89 terminology sets recorded on separate pages of spreadsheets in Japanese were downloaded by the web page of JFMDA [14], and all terms were arranged in one CSV file (Fig. 2). The field names in the CSV file were as follows: “terminology ID,” “medical device problems/patient problems,” “category terms,” “preferred terms,” “synonyms,” “definitions,” and “CDRH-NCIt.” We defined the relationships between terms as follows: (1) a description of the hierarchy relations between category terms and preferred terms using “rdfs:subClassOf,” (2) a description of the relations between preferred terms and synonyms using “hasSynonym,” (3) a description of the relations between preferred terms and CDRH-NCIt terminology using “correspondenceOf.” (Fig. 3). We used Google Refine 2.5 [15] to describe and represent the relations using RDF (Fig. 4).

### Structural inconsistency extraction using SPARQL

The terminology has a two-layer hierarchical structure of category terms and preferred terms. In addition, the definition statement attached to a preferred term has to be unique, the preferred terms should be unified in a terminology set, and the CDRH-NCIt terms must be uniquely determined. In order to evaluate inconsistencies in the integrated terminology, the following six matters were evaluated:

1. Is the two-layer structure between the category and preferred terms consistent without exchanging both terms depend on the terminology items?
2. Are preferred term and a synonym exchanged depend on the terminology items?
3. Is each preferred term subordinate to one category term?
4. Are the definitions of terms uniquely determined?
5. Are CDRH-NCIt terms corresponding to the preferred terms uniquely determined?
6. Is a term in a medical device problem used for a patient problem?

**Fig. 5:**
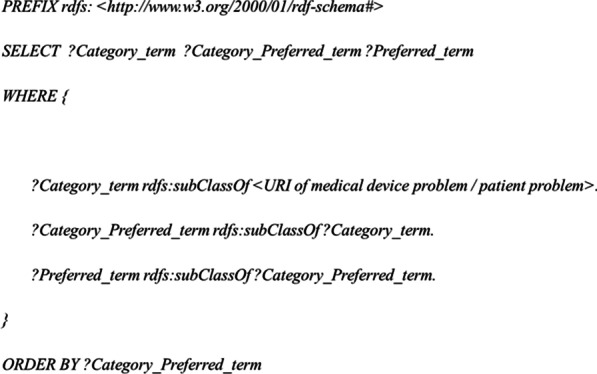
SPARQL query for inconsistency of the hierarchical structure. “Category_and_Preferred_term,” is the term that is present in both the category term and the preferred term. The number of “Category_and_Preferred_term” were aggregated

Detecting the six inconsistencies used SPARQL. In (1), in order to ensure consistency of the two-layer hierarchy in medical device and patient problems, it is not desirable that a term is present in both the category and the preferred terms. Otherwise, when the terminologies are integrated, three or more layers are created. Therefore, it is important to use a query to locate terms that belong to both category and preferred terms. The SPARQL query (Fig. 5) was adopted for the RDF of medical device problems and for that of the patient problems, respectively. The extracted terms that are present in both the category term and the preferred term were aggregated in a spreadsheet.

In (2), the modified query in Fig. 5 was applied to locate a term where a preferred term and a synonym are exchanged by different terminologies. The exchanged terms were aggregated in a spreadsheet. In (3), when a preferred term is subordinate to multiple category terms, it means the definition of the preferred term is not unique. We located the preferred terms which appear in several “category terms” using SPARQL and aggregated the number of the preferred terms. In (4), in order to detect inconsistencies in definition sentences, we extracted the combinations of the preferred terms these definition sentences and identified preferred terms with two or more definition sentences in a spreadsheet. In (5) in order to identify preferred terms with two or more CDRH-NCIt terms, we performed the same steps above. In (6), we detected terms in the sub classes of both “medical device problems” and “patient problems” in the two integrated terminologies.

We used the plugin “sparql-query-plugin-2.0.1” in the ontology editor Protégé 5.1.0 [16]. The correctness of the SPARQL query was verified by comparing with the original data.

## Results

### Summay of the number of terms relations

Before integrating JFMDA terminology, there were 1001 category terms for “medical device problems,” 730 for “patient problems,” 3382 preferred terms for “medical device problems,” and 3382 for “patient problems.” After the terminology integration using RDF, there were 1840 terms for “medical device problem,” and 1629 terms for “patient problem” together with category and preferred terms. The integration removed duplicated terms, a decrease of about 60% in the “medical device problem” and “patient problem.”

Before integrating JFMDA terminology, the numbers of relations in “medical device problems” were: “correspondenceOf” 2765, “hasSynonym” 2525, and “isDefinedBy” 3382. Those of patient problems were 2275, 1362, and 3164, respectively. After merging, duplicates were deleted and the following numbers of terms were obtained: in “medical device problems,” “correspondenceOf” 960, “hasSynonym” 456, and “isDefinedBy” 1250. In patient problems after merging, 667, 356, and 1202, respectively.

### Inconsistency hierarchical structure in the category and preferred terms

As a result of the SPARQL search using query (1), 1457 patterns in medical device problem and 630 patterns in patient problem were found to be inconsistency hierarchies with exchanging category and preferred terms depending on the terminology items. Table 2 shows the examples of the inconsistency hierarchies. Except for duplicates, there were 69 terms (3.5%) in medical device problems which were among both “category” and “preferred terms,” and there were 79 terms (4.8%) in patient problems.

**Table 2:** Examples of unsatisfactory cases of the two-layer structure (English terms in parentheses) “Category and preferred term” is a term which change the allocation depending on the terminology item

	Category term	Category and preferred term	Preferred term
Medical device problems	電気的不良 (electrical defect)	ヒューズ切れ (fuse blown)	ヒューズ溶断 (fuse melting)
	機器不良 (faulty device)	アラーム異常 (alarm abnormality)	異常検知不可 (impossibility of anomaly detection)
	不明 (uncertain)	不明 (uncertain)	原因不明 (cause unknown)
	故障 (defect)	バッテリ不良 (battery problem)	早期放電 (early discharge)
	動作不良 (malfunction)	バッテリ不良 (battery problem)	バッテリ駆動不良 (failure to Run on Battery)
	誤穿刺(erroneous puncturing)	誤穿刺(erroneous puncturing)	誤穿刺(erroneous puncturing)
Patient problems	アレルギー症状 (allergic symptoms)	かゆみ (itching)	そう痒感 (pruritus)
	機能性障害 (functional disorder)	呼吸不全 (respiratory insufficiency)	動脈血酸素飽和度低下 (falling arterial oxygen saturation degree)
	炎症 (inflammation)	痛み (pain)	のどの痛み (sore throat)
	炎症症状(inflammatory symptom)	皮膚炎 (dermatitis)	痒み (itching)
	損傷 (injury)	外傷 (trauma)	損傷 (injury)

“Category term” is a hypernym when “category and preferred term” is described as preferred term. “Preferred term” is a hyponym when “category and preferred term” is described as category term

### Exchanging preferred term and synonym depend on the terminology item

Using query (2), there were 83 patterns of relationships among the “preferred terms,” “preferred terms and synonyms” among medical device problems and 54 patterns among patient problems (Table 3). Except for duplicates, there were 32 terms (1.7%) among medical device problems which were in both “category term” and “preferred term,” and there were 26 (1.6%) terms among patient problems.

**Table 3:** Examples of unsatisfactory cases of preferred terms and synonyms (English terms in parentheses)

	Preferred term	Preferred term and synonym	Synonym
Medical device problems	剥離 (exfoliation)	はがれ (peeling)	剥離 (exfoliation)
	変形 (deformation)	へこみ (cratering)	陥没 (collapse)
	変形 (deformation)	折れ (fold)	折損 (breakage)
	変形 (deformation)	曲がり (bend)	湾曲 (curvature)
	誤作動 (malfunction)	誤動作 (incorrect action)	意図しない動作 (unintentional action)
	挿入不能 (unable to insert)	迷入 (incorrect insertion)	網膜下迷入 (subretinal migration)
Patient problems	意図しない組織損傷(unintentional tissue damage)	裂傷 (laceration)	切り傷 (incised wound)
	残留 (remaining)	遺残 (persistence)	異物残存(foreign matter remaining)
	感染症 (infectious disease)	感染 (infection)	患者感染 (patient infection)
	感染症(infection disease)	感染 (infection)	合併症 (complication)
	感染症 (infectious disease)	感染 (infection)	職業感染 (occupational infection)
	感染 (infection)	感染症 (infectious disease)	感染 (infection)

“Preferred term and synonym” is a term which change the allocation depending on the terminology item. “Preferred term” is a described term in a terminology item when preferred term and synonym” is described as sysnonym. “Synonym” is the opposite

### Preferred terms which have several category terms

Using query (3), the number of preferred terms appearing in two or more category terms was 157 (9.5%) in medical device problem and 171 (10.5%) in patient problem. The maximum number of category terms that one preferred term has was 11 in medical device adverse event. In patient problem, the maximum number was 7. Examples are shown in Table 4.

**Table 4:** Examples of preferred terms which have several category terms (English terms in parentheses)

	Preferred term	Category term
Medical device problems	アーチファクト (artifact)	アーチファクト(artifact), 異常画像 (image abnormality)
	バッテリ不良 (battery defect)	充電不良 (charging defect), 動作不良 (malfunction), 故障(defect)
	劣化 (degradation)	経時変化 (temporal change), 故障 (defect), 破損 (damage), 不良 (failure)
	変形 (deformation)	故障 (defect), 破損 (damage), 成形不良 (shape defect), 変形不良 (deformation defect), コンタクトレンズ不良 (defect of contact lenses), 分注ノズル折損・変形 (breakage and deformation in dispensing nozzle), 機械的不良 (mechanical failure), 不良 (failure), 損傷 (damage), 意図しない効果 (unintentional effect), 品質不良 (poor in quality)
Patient problems	かぶれ (irritated skin)	炎症症状 (inflammatory symptom), 炎症 (inflammation), 皮膚炎 (skin inflammation), かぶれ (irritated skin)
	骨折 (bone fracture)	骨折 (bone fracture), 外傷 (trauma), 損傷 (injury), 組織損傷 (damage to tissue), 組織障害 (tissue involvement)
	神経障害 (nerve disorder)	組織損傷 (damage to tissue), 機能性障害 (functional damage)
	失明 (no light perception)	視機能障害 (damage of visual function), 視力障害 (visual disorder), 失明 (no light perception)
	アレルギー反応 (allergic reaction)	感染 (infection), 炎症 (inflammation), ショック (shock), アレルギー症状 (allergic symptoms), 眼疾患 (eye disease), 損傷 (damage), アレルギー反応 (allergic reaction)

### Definitions of terms that have multiple definitions

Using query (4), the preferred terms which have two or more definitions were 155 (8.4%) in medical device problem and there were 161 (9.9%) in patient problem. The adverse event term with the largest number of statements was “緩み(slack)” which had 10. In patient problem, “感染 (infection)” had 8 definitions and that was the largest number of all the preferred terms (Table 5).

**Table 5:** Examples of terms with multiple definitions (English terms in parentheses)

	Preferred term	Definition
Medical device problems	アーチファクト (Artifact)	信号処理などで、観測や解析の段階で発生したデータのエラーや信号のゆがみが画像に混入すること。 (Appearance of data error and deformation of images after image processing)
		目的としない信号が表示されること (Displaying unwanted signals)
	ヒューズ切れ (fuse blow)	ヒューズが切断すること。 (Fuse blows)
		過電流等によりヒューズが切れること (Fuse blown due to overcurrent)
	バッテリ不良 (defective battery)	バッテリ電圧・容量の低下 (Low battery voltage and capacity)
		バッテリの不良 (battery defect)
		バッテリ自体の不良 (battery defect itself)
		バッテリの異常でバッテリによる駆動ができないこと。 (A device cannot be driven due to a battery failure.)
	緩み (slack)	ネジ、ロック等が緩むこと。 (Loose screws, locks, etc.)
		ねじなどの締め付けが甘くなること。 (A screw has become loose.)
		ネジ等が緩み、当該部位の物理的な保持・作用に支障をきたす状態の事 (A condition in which screws or other parts are loosened and interfere with the physical retention and operation of the relevant part)
		構成部品の固着部 (ねじ、接着等) の結合力が弱まり、正常状態に比べて部品が動いてしまうこと (The bonding force of the fixed parts (screw, adhesive, etc.) of the component parts is weakened, and the parts move incorrectly compared to the normal state)
		接続不良となる接続部のがたつき。 (Loose connections, causing connection failure)
		接続部分、固定などが緩むこと。 (Loose connections, fixings, etc.)
		装置又は器具の部分の固定が不良となること。 (Insufficient fixing of parts of equipment or instruments)
		通電不良となる接続部の固定力不足 (Insufficient fixing force of the connection part that results in poor conduction)
Patient problems	感染症 (infection disease)	ポケット部位から感染症となる。(Infections from pockets)
		病原体が生体内に侵入し、一定の病変を惹起すること。 (The bacteria enters the body, and causes certain lesions.)
	心室細動 (Ventricular fibrillation)	心室が整合的な収縮を行なわず、各部の筋肉が無秩序に収縮する状態 (The ventricle does not pump normally, and the muscles in each part pump randomly)
		心臓の心室が小刻みに震えて全身に血液を送ることができない状態。 (A state in which the ventricle of the heart trembles and cannot send blood to the whole body)
		心室が規則に震えるように痙攣 (けいれん) する状態のこと。 (A state in which the ventricles convulse so as to tremble according to rules)
		患者の心臓の拍動が小刻みな状態となって、拍出ができない状態。 (A state in which the patient's heart quivers, or fibrillates and cannot pump blood)
		悪性の心室不整脈で、心室筋の持続的、非協調的な収縮によって特徴づけられ、心臓からの血流は途絶する。 (A malignant ventricular arrhythmia, characterized by sustained, uncoordinated contractions of ventricular muscle, disrupting blood flow from the heart.)
	手術時間の延長 (Extended operating time)	医療機器の不具合により当初予定していたよりも手術時間が長引いてしまうこと。 (The operation time is longer than originally planned due to an adverse event of medical equipment.)
		医療機器の使用にともなう健康被害の発生に関連し、手術時間が長引いてしまうこと。 (Prolonged operation time associated with the occurrence of patient problems associated with the use of a medical device)
	虹彩脱出 (Iridocele)	眼球の穿孔部からの虹彩の一部が眼球外に脱出した状態。強角膜の穿孔性外傷や切開手術、または角膜軟化症・角膜炎が進行して角膜が穿孔したときに生じる。感染防止が重要で、速やかな虹彩整復手術を要する。 (A state in which part of the iris from the perforated part of the eyeball has escaped outside the eyeball. It occurs when corneal perforation occurs due to progression of sclerocorneal perforating trauma or incision surgery, or corneal malacia/keratitis. It is important to prevent infection and conduct immediate iris reduction surgery.)
		眼球の穿孔部からの虹彩の一部が眼球外に脱出した状態 (Part of the iris from the perforated site of the eyeball prolapse outside the eyeball)
		虹彩が脱出すること。 (Prolapse of the iris)
	感染 (Infection)	病原微生物が人体に侵入し、臓器や組織中で増殖し、種々の症状をもたらすこと (Pathogenic microorganisms invade the human body, multiply in organs and tissues, and cause various symptoms.)
		病原微生物が人体に侵入し、臓器や組織中で増殖し、種々の症状をもたらすこと。 (Pathogenic microorganisms invade the human body and multiply in organs and tissues, causing various symptoms.)
		微生物が、体内に侵入・定着した状態。 (A state in which microorganisms have invaded and settled in the body)
		微生物が人体に侵入、増殖、何らの症状が出現する症例 (Cases in which microorganisms invade the human body, proliferate, and manifest any symptoms)
		病原微生物または感染性物質が人体に侵入し、臓器や組織中で増殖し、種々の症状をもたらすこと (Pathogenic microorganisms or infectious substances can enter the human body, multiply in organs and tissues, and cause various symptoms.)
		微生物感染などによるもので、キズ口の周りが赤くなっていたり、ズキズキした痛みが続いたり、膿を持っていたり、熱や腫れ等の異常が認められる場合 (Due to microbial infection, etc., when the area around the wound is red, the throbbing pain continues, there is pus, or there are abnormalities such as fever or swelling.)
		微生物が人体に侵入、増殖、何らの症状が出現する症例。 (A case in which microorganisms invade the human body, proliferate, and show any symptoms)
		微生物が、体内に侵入・定着した状態をいう。(A state in which microorganisms have invaded and settled in the body)

### CDRH-NCIt terms corresponded to more than two preferred terms

Using query (5), the number of preferred terms which include two or more CDRH-NCIt terms were 160 (8.7%) in medical device problem and 95 (5.8%) in patient problem. The adverse event term with the largest number of CDRH-NCIt terms was 9 with “変形 (deformation).” In patient problem, “穿孔 (infection)” it was 4 CDRH-NCIt terms, and this was the largest number among the preferred terms (Table 6).

**Table 6:** Example of preferred terms which have more than two “CDRH-NCIt terms” (English terms in parentheses)

	Preferred term	CDRH-NCIt term
Medical device problems	ねじれ (twisted)	Material twisted, kinked
	アラーム誤作動 (defective alarm)	Defective alarm, improper alarm, device alarm system issue
	アラーム音不良 (alarm sound failure)	Device alarm system issue, not audible alarm
	変形 (deformation)	“Lens aberration, distortion of,” material distortion,
		“Quality, unsatisfactory or poor,”
		Material deformation, “component(s), broken,”
		Material deformation material deformation,
		bend, material integrity issue
Patient problems	巨大乳頭結膜炎 (giant papillary conjunctivitis)	Foreign body reaction, hypersensitivity, conjunctivitis
	湿疹 (eczema)	Rash, skin inflammation
	アレルギー反応 (allergic reaction)	Hypersensitivity, allergic reaction
	穿孔 (perforation)	“Vessels, perforation of,” cardiac perforation, perforation, injury

### Terms among medical device problem included among patient problems

Using query (6), there were 8 terms which appeared in both medical device problem and patient problem. Table 7 shows the differences between the definition in medical device problem and in patient problem.

**Table 7:** Differences between the definition in medical device problem and in patient problem

Term	Term concept in medical device problem and patient problem
損傷 (damage/injury)	(medical device problem) Break
	(patient problem) Injury
汚染 (contamination)	(medical device problem): Contamination of device
	(patient problem): Contamination of patient
発熱 (fever)	(medical device problem) generation of heat from a device or its component
	(patient problem) Fever
感染 (infection)	(medical device problem) An aseptic condition cannot be maintained due to broken packaging, etc. Exists in the category term
	(patient problem) Bacteria invade the human body
感電 (electrification)	(medical device problem/patient problem) get an electric shock
その他の事象 (other events)	(medical device problem/patient problem) This term exists as a category term and preferred terms which are not allocated in other category terms are involved
その他 (Others)	(medical device problem) There was only one combination of the category term “Others” and the preferred name “Others”. It was defined as “an unknown medical device problem.”
	(patient problem) Below the category term “others,” three preferred names exist: “others,” “unknown,” and “no patient problems.”
不明 (unknown)	(medical device problem) cause unknown
	(patient problem) cause unknown and no information

## Discussion

In this study, we integrated 89 items of terminology using RDF, and identified inconsistencies in the hierarchical structure, the relationship between synonyms and preferred terms, and the definition statements using SPARQL. About 60% of the total number of duplicated terms were found. This is because industry associations that created multiple terminologies adopted the same terms in terminologies of similar medical device groups. In the case that all terms with the same spelling have the same concept, efficient integration can be achieved automatically using RDF. Furthermore, we evaluated six matters of inconsistency in this study, terms that need to be reviewed terms accounted for about 10% or less than 10% in each item. It may take a lot of effort to detect these from thousands of words if it is done manually. Since SPARQL can do this automatically, it has the advantage of reducing the physical and time burden.

### Inconsistency of two-layer hierarchies

In inconsistency hierarchical structure, 1457 patterns were found among medical device problems and 630 patterns among patient problems using SPARQL. Inconsistencies of two-level hierarchies can be approximately divided into four kinds: (1) category terms which indicate the schema and preferred terms which indicate the details such as the relationships between “faulty device” and “alarm abnormality” (Table 2), (2) the relationship between a category term and a preferred term as a cause-effect relationship, such as the relationships between “battery problem” and “early discharge” (Table 2) among medical device problems, and between “respiratory insufficiency” and “falling arterial oxygen saturation degree,” (3) the hierarchy levels of category terms and preferred terms are reversed (inverted) depending on the terminologies [For example, in one terminology “trauma” is listed as a category term (hypernym) and “injury” as a preferred term (hyponym), but in another terminology they are reversed], and (4) the same words are listed as category terms and preferred terms, such as for “erroneous puncturing” and “uncertain”.

In terminology development, the relationship between hypernym and hyponym is “is-a relation.” The relationship is reflexive and transitive, but not symmetric [17]. (1) is applicable to this rule. The reason why more than three levels are structured is that the granularity of the terms differs depending on industry associations. It is necessary to consider unifying the granularity among the industry associations. In (2), depending on the medical device manufacturer, it may be easier for users to describe the relationship between the category term and preferred term as a causal relationship rather than an inclusive relationship. However, when integrating multiple terminologies, if the inclusion and causal relationships are mixed, the preferred term belonging to the category term becomes inconsistent, which may cause difficulties for the user searching for a term. Accurate aggregation may also be hindered. It is necessary to request the industry association that created the term in which the relationships between the category terms and the preferred terms have a causal relationship to reconfirm the hierarchical structures they are basing it on, or to correct the term using a tool that classifies mechanically. In (3), the inversion of hypernyms and hyponyms means that they are homonymy. If it is correct, it is desirable to consider unifying the notation, and if not, consider unifying the order of hypernyms and hyponyms, or describing another notation. In (4), when developing the terminology using the bottom up method, it is considered that the category terms and the preferred terms became the same because there may have been no appropriate category terms. In addition, these may be autohyponyms [18]. Autohyponym indicates that the hyponym is a subset of the hypernym. In this case, unless exploring a different more appropriate notation or considering managing terms by ID number, there will be discrepancies in the hierarchical structure because the same notation is regarded as the same term by RDF.

### Polyhierarchy

Some terminologies have adopted polyhierarchy. In SNOMED-CT, a subtype hierarchy is a directed acyclic graph [19]. Cimino described that general consensus seems to favor allowing multiple hierarchies to coexist in a vocabulary and one could be so designated with the others treated as nonhierarchical with directed and acyclic relationships [20]. It would be possible if JFMDA also became a valid directed acyclic graph when integrated consistently. However, some parts of this integrated terminology were cycle graph due to the inconsistency of the hierarchical structure and the cycle graph between two words as shown in (7).

One of the features of the preferred terms having a number of category terms among medical device problems expressed the cause of the category terms. For example, “battery defect” as the preferred term expressed the cause of “charging defect,” “malfunction,” and “defect” (Table 4). Among patient problems, the tendency of the relationship between category terms and the preferred terms is a pattern of cause-effect relation, such as “trauma” in the category term and “bone fracture” in the preferred term (Table 4). The relationship between category terms and the preferred terms is the opposite compared with that of medical device problems. Since there is a possibility that two terms that each industry association considers to be related are set as hypernyms and hyponyms, it will be necessary to request the industry association to make corrections. Although the JFMDA terminology has been developed based on a monohierarchy, it is necessary to consider allowing a multi-layered structure with directed acyclic graph while accepting differences in industry ideas.

### Preferred term and synonym

There were 32 terms among medical device problems which appeared in both the category term and preferred term, and there were 26 terms in patient problem. The following three kinds were found: (1) according to the terminology, preferred terms and synonyms are opposite, such as “exfoliation” (剥離) and “peeling” (はがれ), (2) preferred terms and synonyms are connected by causal relationships, such as “deformation” (変形) and “cratering” (へこみ), and (3) preferred terms and synonyms seem to be connected by “is-a relation,” such as “aberrance” (迷入) and “subretinal migration” (網膜下迷入).

In (1), it should be unified to one or the other. In (2), “cratering”, “fold”, and “bend,” which are synonyms associated with “deformation,” and these terms mean the causes of the “deformation.” The term “deformation” includes various concepts from the views of different industry associations, therefore it may be interpreted differently depending on the industry association. It is preferred that spellings which describe the details are used as preferred terms and the others are adopted synonyms. “Deformation” should not be used as the preferred name, and “cratering”, “fold”, and “bend” are preferred as preferred terms instead. “Collapse,” “breakage,” and “curvature” should be used as synonyms. In (3), it is considered that the terms were used by omitting the part of spellings in some terminology fields. It is necessary to use the spellings expressed in detail as preferred terms in order to carry out accurate statistical analysis as above.

### Definition statements

There were some patterns of definition statements. First of all, as patterns common to medical device problem and patient problem, whether there are punctuation marks or not. Secondly, various expressions were used to describe the same concept, such as “a state in which the ventricle of the heart trembles” and “convulsions” in “ventricular fibrillation.” Additionally, as to the specific pattern in medical device problem, some definitions included the cause of the adverse event and the others did not include it, such as “blown fuse.” As to the specific pattern in patient problem, one is whether the cause of the patient problem is included or not as well as the medical adverse event, another is the cause of the patient problem is different, such as “extended operation time,” the other is whether to include a countermeasure for patient problem or not, such as “iridocele.” Those who use the JFMDA terminology may be confused if multiple definitions are given to one term. Therefore, if plural definition statements express the same concept, they should unify the description. If not, constructors should use different terms.

### Multilingual mapping

There are international terminologies for medical devices related to adverse events, especially IMDRF, a voluntary group of medical device regulators from around the world that has constructed the terminology to accelerate international medical device regulatory harmonization and convergence [2]. Multilingual terminology mapping is an important process of finding correspondences between terminologies in different languages to allow these to be mutually understandable.

In JFMDA terminology 1st edition, mapping between the preferred terms and CDRH-NCIt terms was conducted manually by the industry associations. The result of an inconsistency detection survey using SPARQL showed that there were 160 preferred terms in JFMDA which appear in two or more CDRH-NCIt terms in medical device problems, and there were 95 among patient problems. This may be due to differences in the interpretation of CDRH-NCIt terms among industry associations. However it is also possible that the Japanese concept and the English concept do not exactly match. In ontology, there are two main strategies for alignment: direct and indirect alignment. The direct alignment is translation-based and uses external resources to help with the translation, while the indirect alignment uses intermediary mapping between the source and target ontologies. In addition, mapping two ontologies can be an automated or manual process [21]. Manual mapping is still the most common choice, but it necessitates a large team of experts; it is time consuming and prone to errors. Meanwhile, automated methods use publicly available terminology resources, but the sources of these are largely incomplete outside of the English speaking world [21]. To improve inconsistencies, it is being considered to reduce the human and time resources required and ensure accuracy using the following process: translating the international (not available in Japanese) terminology into Japanese, performing automatic mapping using machine learning, and confirming the results manually.

### Whether a term among medical device problems is included in patient problems

There were 8 terms which are present among both medical device problems and patient problems. These terms share the concept, but depending on the situation where they are used, the subject can be the device or the patient. Therefore, this term was included among both medical device problems and patient problems. However, if the same notation is used, there is a problem that the hierarchies of terms in medical device problem, and those in patient problem are exchanged in mapping by RDF. Therefore, it is necessary to change the notation such as “damage (medical device problem)” and “damage (patient problem)” to distinguish both.

## Limitations and future work

A previous study [4] identified six problems in terminologies: incorrect schemas, misunderstanding of semantics of attributes, incomplete modelling, over-literal definitions, not tracing errors to their roots, and lack of normalisation. “Incorrect schemas,” “misunderstanding of semantics of attributes” and “not traced, in detecting structural problems” correspond to polyhierarchy in this study. “Incomplete modeling” corresponds to the determination of preferred terms with multiple definition statements. For the other problems, it is necessary to perform an analysis considering the concepts of the terms and definition statements. However, it is difficult to do this using RDF and SPARQL. In synonym determination, we are conducting research using edit distance and distributed representation using definition statements of preferred terms [22]. In addition, a rule-based method for detecting synonyms from word notation has also been proposed [23]. We believe that applying these technologies will work effectively to improve the inconsistencies in terminology mapping.

## Conclusions

In this study, we integrated JFMDA terminology and identified 6 items of inconsistencies. The RDF and SPARQL are useful tools to explore inconsistencies of hierarchies, definition statements, and synonyms when integrating terminology by term notation automatically. As future work, we will consider a method which can take into account concepts of terms in order to improve the inconsistency detection method.

## Data Availability

The data that support the findings of this study are available from The Japan Federation of Medical Devices Associations (jfmda.gr.jp/activity/committee/fuguai/).

## Abbreviations

CDRH-NCIt: The centers for devices and radiological health and the national cancer institute’s thesaurus
IMDRF: The International Medical Device Regulators Forum
ISO TC: The International Organization for Standardization Technical Committees
JFMDA: The Japan Federation of Medical Devices Associations
RDF: Resource Description Framework
SPARQL: SPARQL Protocol and RDF Query Language
